# Tongue coating in relationship to gender, plaque, gingivitis and tongue cleaning behaviour in systemically healthy young adults

**DOI:** 10.1111/idh.12416

**Published:** 2019-10-10

**Authors:** Laura M. Van Gils, Dagmar E. Slot, Eveline Van der Sluijs, Nienke L. Hennequin‐Hoenderdos, Fridus (GA) Van der Weijden

**Affiliations:** ^1^ Department of Periodontology, Academic Centre for Dentistry Amsterdam (ACTA) University of Amsterdam and Vrije Universiteit Amsterdam The Netherlands

**Keywords:** bleeding, cross‐sectional study, gingivitis, oral hygiene behaviour, plaque, tongue coating thickness, tongue surface discoloration

## Abstract

**Objectives:**

The purpose of this observational study was to investigate the relationship between tongue coating (thickness [Tc] and surface discoloration [Td]) and gender, plaque, gingivitis (bleeding on marginal probing [BOMP] and bleeding on pocket probing [BOPP]) and tongue cleaning behaviour.

**Materials and Methods:**

A total of 336 participants were screened for this cross‐sectional study, from which 268 (150 male, 118 female) were found to be eligible. Aspects of tongue coating were visually assessed. Additionally, BOMP, BOPP and the plaque index (PI) were scored. To ascertain the tongue cleaning behaviour, the Oral Hygiene Behavior questionnaire was used.

**Results:**

Most tongue coating was found at the posterior sections of the tongue surface. A thin coating and white discoloration were most prevalent as highest score for both males (92.7%) and females (87.4%), as well as white discoloration for the whole group of participants (50.2%). A gender difference was observed for TC and Td (*P* < .001). Analysis did not reveal a relationship between Tc and PI and between Td and PI. Also, no relation was detected between tongue cleaning behaviour and Tc or Td. However, tongue cleaning was associated with lower BOMP and BOPP scores.

**Conclusion:**

BOMP, BOPP or PI score did not appear to be linked to Tc and Td. A significant gender difference was found for Tc and Td. Self‐reported tongue cleaning behaviour was associated with slightly lower BOMP and BOPP scores.

## 1 INTRODUCTION

The tongue occupies about a third of the surface area of the oral cavity. Additionally, the papillary structure of its dorsum makes it the largest oral surface and favours the accumulation of small particles. Consequently, the tongue dorsum harvests mostly oral microbes that aggregate with detached epithelial cells, food, and saliva and serum components, forming a layer of so‐called tongue coating.^1^, ^2^ The formation of tongue coating is a normal phenomenon in health, where most coating is found on the posterior third of the tongue.^3^

As oral microbes dictate health and disease, it is not surprising that the densely‐populated tongue dorsum influences the oral ecosystem. For instance, the microbial composition of saliva stimulated by chewing is very similar like the tongue dorsum,^4^ and the pH of the oral cavity has been associated with the appearance of the tongue.^5^ Furthermore, tongue coating is increased in periodontal disease.^3^, ^6^ Also, periodontitis‐associated bacteria present in tongue coating have been closely associated with those in dental plaque.^7^, ^8^ Therefore, it is likely that the bacteria in the coating of the tongue act as a reservoir via the saliva for those in the plaque biofilm on the teeth.^9^

Compared to the gums and teeth, the tongue has not received much attention from dental researchers. As a consequence, there are relatively few clinical studies describing tongue coating, especially in health. Mantilla Gómez and colleagues in 2001 used a detailed assessment to describe the Tc and Td in 70 healthy/gingivitis participants.^3^ They included participants within a wide age range but in their analysis showed that age was a significant factor with respect to the prevalence of tongue coating. They also did not record whether participants habitually cleaned their tongue, while it is known that this may impact the surface appearance.^10^ Also, other oral hygiene habits such as tooth cleaning and interdental cleaning may impact tongue coating.^11^

For the present study, a cohort of 268 non‐smoking systemically healthy young adults, within a relatively small age range, was evaluated for tongue surface appearance (ie coating and discoloration). Subjects were questioned about their oral hygiene habits, including tongue cleaning. In addition, the relationship with the level of dental plaque, gingivitis and gender was assessed.

## 2 MATERIAL AND METHODS

### 2.1 Ethical procedures

This study received the approval of the medical ethics committee of Academic Medical Centre of Amsterdam (2012_210#B2012406), registered at the Dutch Trial Register (NTR3649) and was conducted in accordance with the Declaration of Helsinki (2008) of the World Medical Association and approximating Good Clinical Practice guidelines. All participants signed an informed consent form. This observational clinical study was performed at the Academic Centre for Dentistry Amsterdam, The Netherlands, within a framework of the Top Institute Food and Nutrition as part of the project ‘Estimating the boundaries for a healthy oral ecosystem in young individuals’.

### 2.2 Participants and study design

The study population contained a convenience sample of 268 systemically healthy young adults, 18‐32 years of age with an average of 22.6 years, without periodontitis. The screening of suitable volunteers took place from October 2012. Participants were selected, based on their health status assessed by a medical questionnaire, if they had visited their general dentist the previous year and were regarded to be without oral or dental problems. Periodontal screening was performed according to the criteria of the Dutch Periodontal Screening Index (DPSI).^3^, ^12^ (for details see online supplement Table 1). The inclusion criterion was a DPSI ≤3 minus, which corresponds to:

Score 0: No pockets >3 mm, no calculus, no overhanging restorations, no bleeding on pocket probing,
Score 1: The same criteria as score zero but with presence of bleeding on pocket probing,
Score 2: The same criteria as score one but with the calculus and/or overhanging restorations
Score 3‐: A maximum probing depth of 4‐5 mm in the absence of gingival recession.

The enlisting protocol, exclusion and inclusion criteria are described in detail in previous studies.^13^, ^14^ Particularly, exclusion criteria were: smoking, presence of systemic disease; overt dental caries; oral infections; recent use of antibiotics; and use of anti‐inflammatory drugs or other prescribed medication (except for oral contraceptives) which could interfere with the outcome of this study.

### 2.3 Questionnaires

Participants were instructed not to eat, drink, chew gum, or perform strenuous physical exercise before the appointment, and to refrain from oral hygiene procedures 24 hours before their appointment. In addition participants were instructed to refrain from eating and drinking starting from midnight the day before the appointment. For each individual, the assessments took place in one single day in the following order.

#### 2.3.1 Gender and menstrual phase

Participants were asked to record their gender. Females were questioned in which phase of their menstrual cycle they were at the moment of the appointment. The menstrual cycle phase (menstrual, follicular or luteal) was noted for female participants.^14^ The follicular phase was a summary of the follicular and proliferative phase.

#### 2.3.2 Tongue cleaning behaviour

To ascertain the tongue cleaning behaviour of the participants the Oral Hygiene Behavior questionnaire was used.^15^, ^16^ The participants were questioned by one member of the research team (EVDS). The questionnaire was completed together with the participant to assure that all questions were answered. Only closed questions were implemented, which included brushing frequency, brushing time, tongue cleaning and interdental cleaning behaviour frequency such as the rate of floss, woodsticks and interdental brushing.

### 2.4 Bleeding on marginal probing (BOMP) score

The level of gingival inflammation was assessed according to the BOMP‐score index (by EM)^13^ (see online supplement Table 2). The measurement of bleeding as indication of gingival inflammation was recommended by several studies.^17^, ^18^, ^19^ The measurements were scored in the 1st & 3rd quadrants or the 2nd & 4th quadrants. For each participant, these quadrants were randomly assigned. In the opposing quadrants, the dental plaque scores were carried out to avoid potential influence of this assessment on the BOMP scores.

#### 2.4.1 Tongue surface assessment

The tongue surface was visually assessed by one trained examiner (EVDS).^3^ In short, the tongue was divided into 9 sections (3 posterior third, 3 middle third and 3 anterior third) (see online supplement Figure 1.) and each section received two scores: one for Tc (0‐2) (Table 2) and one for Td (0‐4). For Tc, ‘no coating’ was scored when the pink colour of the tongue was visible through the coating, and ‘heavy‐thick coating’ was scored when there was no pink colour visible under the coating. If a section contained a third or more coating or discoloration, it was given the most prevalent score.

**Table 1:** Study demographics and a summary of the clinical parameters divided by gender

	Men N = 150	Women N = 118	Overall N = 268
Mean age (SD) (Min‐Max)	22.2 (2.7) (18‐32)	22.31 (2.6) (17‐30)	22.6 (2.7) (18‐32)
Mean plaque score (SD) (Min‐Max)	1.06 (0.39) (0.15‐2.07)	0.87 (0.40) (0.02‐2.10)	0.98 (0.41) (0.24‐2.10)
Mean bleeding on marginal probing score (SD) (Min‐Max)	0.41 (0.26) (0.01‐1.24)	0.35 (0.21) (0.01‐1.00)	0.38 (0.24) (0.01‐1.24)
Mean bleeding on pocket probing score (SD) (Min‐Max)	0.54 (0.16) (0.22‐0.92)	0.48 (0.15) (0.13‐0.86)	0.51 (0.15) (0.13‐0.92)
Mean tongue coating thickness sumscore^a^ (SD) (Min‐Max)	2.83 (1.76) (0.00‐10.00)	2.23 (1.77) (0.00‐8.00)	2.57 (1.79) (0.00‐10.00)
Mean tongue discoloration sumscore^a^ (SD) (Min‐Max)	5.83 (2.75) (1.00‐14.00)	3.36 (2.12) (0.00‐10.00)	5.33 (2.69) (0.00‐14.00)
DPSI score^b^			
0	0%	0%	0%
1	2.0%	5.0%	3.3%
2	26.5%	40.3%	32.7%
3−	70.9%	53.8%	63.6%

Abbreviations: BOMP, bleeding on marginal probing score; BOPP, bleeding on pocket probing score; DPSI, Dutch Periodontal Screening Index; PS, plaque score; SD, standard deviation; tc, tongue coating thickness score; td, tongue surface discoloration score.

a The mean of the sum of the scores found at the 9 sections in each individual for tongue coating thickness and tongue surface discoloration.

b Percentage subjects with highest score. None of the participants had DPSI score 3 + and 4 since this was an exclusion of this study.

**Figure 1:**
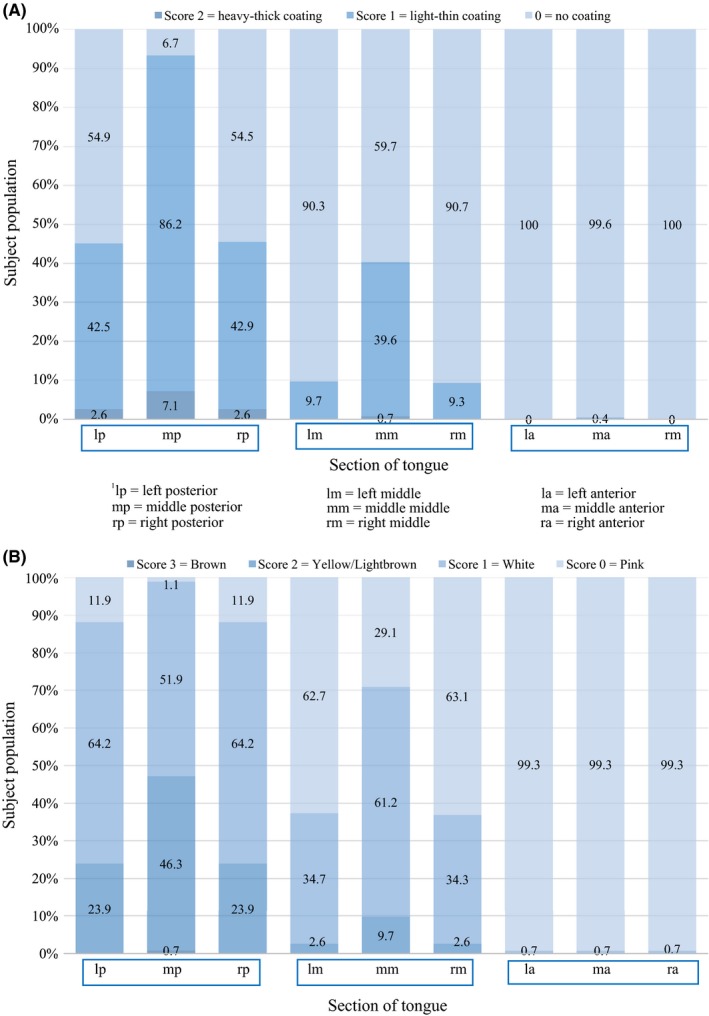
A, Distribution in percentages of the thickness of tongue coating scores according to Mantilla Gómez et al^3^separated for each of the 9 sections of the tongue (see online supplement Figure 1.) (N = 268). B, Distribution in percentages of tongue surface discoloration scores according to Mantilla Gómez et al^3^ separated for each of the 9 sections of the tongue (see online supplement Figure 1.) (N = 268)

#### 2.4.2 Bleeding on pocket probing (BOPP) score full mouth

A full mouth assessment of the level of gingival inflammation was performed according to the BOPP score (by EVDS)^13^ (see online supplement Table 2).

#### 2.4.3 Dental plaque (PI) scores

Dental plaque levels were scored (by DE) at six surfaces on each tooth as is suggested for the modified Silness & Löe index.^20^, ^55^ The plaque score (PI) measurements were scored in randomly assigned the 1st & 3rd quadrants or the 2nd & 4th quadrants.

### 2.5 Statistical analysis

The analyses were performed using SPSS Statistics 23.0 software (IBM). To assess the normality of the data a Kolmogorov‐Smirnov test was used. BOMP, Tc and Td were not normally divided but skewed towards the left. The data of PI and BOPP were normally divided. As most of the data were not normally distributed, non‐parametric tests were utilized.

**Figure 2:**
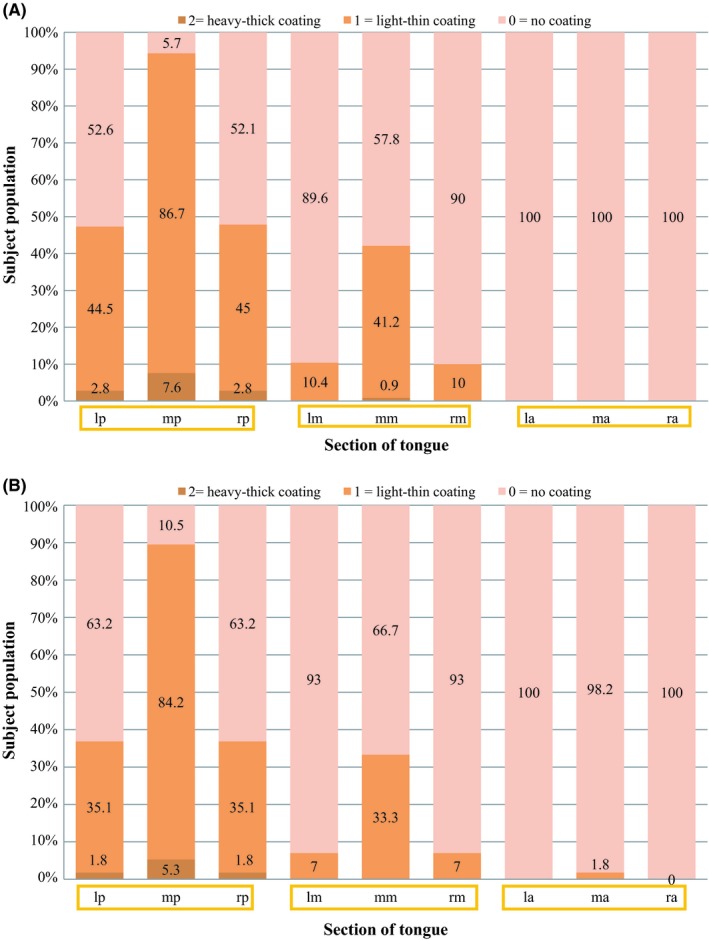
A, Distribution of thickness of tongue surface coating scores according to Mantilla Gómez et al^3^ separated for each of the 9 sections of the tongue (see online supplement Figure 1) for only those participants who mentioned being categorized as ‘never or sometimes tongue cleaners’ (N = 211) (Table 3). B, Distribution of thickness of tongue coating scores according to Mantilla Gómez et al^3^ separated for each of the 9 sections of the tongue (see online supplement Figure 1.) for ‘daily tongue cleaners’ (N = 57) (Table 3)

**Figure 3:**
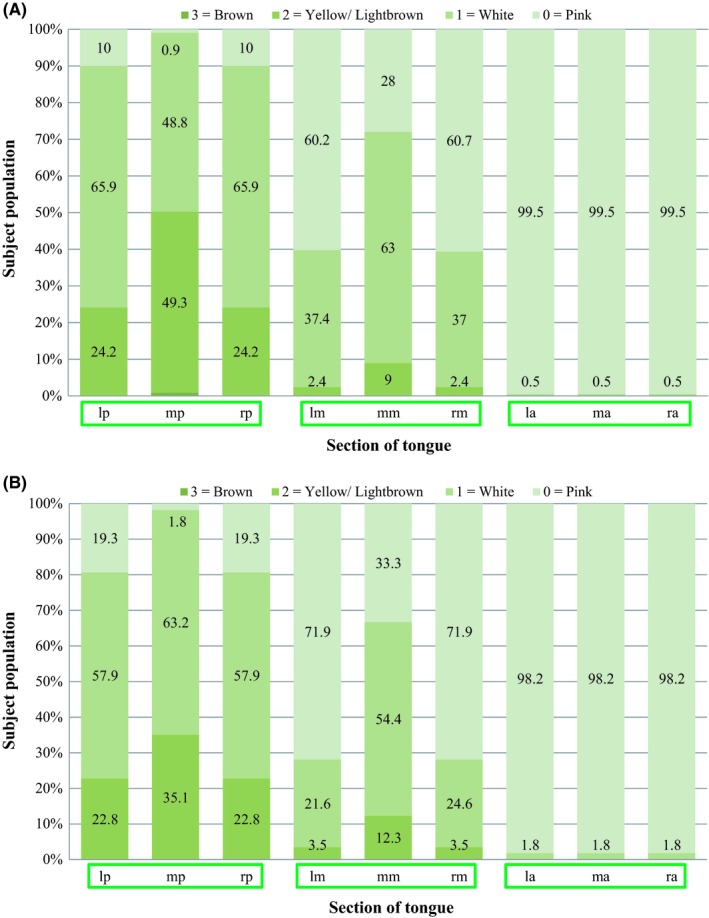
A, Distribution in percentages of tongue surface discoloration scores according to Mantilla Gómez et al^3^ separated for each of the 9 sections of the tongue (see online supplement Figure 1.) for only those participants who mentioned being categorized as ‘never or sometimes tongue cleaners’ (N = 211) (Table 3). B, Distribution in percentages of tongue surface discoloration scores according  to Mantilla Gómez et al^3^ separated for each of the 9 sections of the tongue (see online supplement Figure 1.) for only those participants who mentioned being categorized as ‘daily tongue cleaners’ (N = 57) (Table 3)

Spearman's correlation was used to explore the correlation between various parameters (BOMP, BOPP, PI and Age) and Tc and Td. In all analyses Tc and Td were assessed using the sum of the scores as observed on the dorsum of the tongue in accordance with Winkel et al,^21^ and Van der Sluijs et al^22^ (so a score from 0 to 18 was possible). Tongue cleaning behaviour was divided into two categories: never or sometimes combined compared to daily tongue cleaning Figure 2 and Figure 3. Correlation coefficients were interpreted taking into account the determined values^23^ (see online supplement Table 3). The Mann‐Whitney U test was used to analyse a relationship of gender and tongue cleaning behaviour with Tc and Td.

A sub‐analysis was performed to investigate whether gender, and hormonal change is related to Tc and Td. The relation between Tc, Td and the menstrual cycle of female subjects was evaluated using a Kruskal‐Wallis Test. Due to the multiple comparisons among tongue coating and discoloration the Bonferroni corrections were applied by dividing the critical *P*‐value (.05) by the number of comparisons.

## 3 RESULTS

The experimental period started in October 2012 and was finished in March 2013. In total 336 participants were screened, out of which 268 eligible individuals were examined.^13^ The data of these 268 individuals were analysed. The number of males was 150 (56% of the participants) and the number of females was 118 (44% of the participants). The average age was 22.6 years, with a range of 18‐32 (95% Confidence Interval, 95%CI: 22.7‐23.3). Table 1 shows a description of the study population by mean scores. Among the 268 participants the highest DPSI score was distributed as follows: score 0‐(0%); score 1‐(3.3%); score 2‐(32.7%) and score 3‐(63.6%).

### 3.1 Tongue surface assessment

Figure 1a shows the distribution of the prevalence of Tc scores on the dorsum of the tongue. Most of the coating is found at the posterior section of the tongue where the mid posterior section shows a higher prevalence of heavy‐thick coating (7.1%) whereas on the anterior section of the tongue less coating was found (0% coating on the right and left anterior section of the tongue and 0.4% light‐thin coating was found on the middle anterior section). Figure 1b shows the distribution of the prevalence of Td on the dorsum of the tongue. Also, a large part of discoloration is found at the posterior aspect with the mid posterior section showing the highest prevalence of yellow coating (46.3%). This section is also the part of the tongue where brown discoloration was observed. The anterior section of the tongue contains the least amount of discoloration (0.7% white coating). Table 2 shows the maximum percentages of Tc and Td summarized by data of all 9 sections, distinguishing between male and female participants.

**Table 2:** Maximum percentages of tongue coating thickness and tongue surface discoloration divided by gender presented by percentage of subjects, one or more of the 9 sections showing this aspect as highest score for the total tongue surface

	Male N = 150 (%)	Female N = 118 (%)	Overall N = 268 (%)
Tongue coating thickness			
0 = no coating	0.7	3.4	1.9
1 = thin coating	92.7	87.4	90.7
2 = thick coating	6.0	8.4	7.1
Tongue surface discoloration			
0 = no discoloration	0	1.7	0.7
1 = white discoloration	41.1	61.3	50.2
2 = yellow/ light brown discoloration	57.0	36.1	48.0
3 = brown discoloration	1.3	0	0.7

### 3.2 Oral Health Behaviour

Results of the questionnaire regarding Oral Hygiene Behavior are shown in Table 3, where frequencies of the answers of participants are presented. All 286 participants completed the questionnaire (N = 286), but 15 respondents could not decide between the usage of a power toothbrush or a manual toothbrush because of hybrid use. So, they answered this question choosing both options. The majority of the participants use a manual toothbrush and brush twice a day for 2 minutes. Most of the participants never use woodsticks, interdental brushes or floss.

**Table 3:** Oral hygiene behavior including tongue cleaning behavior (N = 268)

Question	Answer	Number of participants	Percentage of participants (%)
What kind of brush do you use?	Manual toothbrush	185	69.0
	Power toothbrush	98	35.6
How often do you brush?	Not every day	1	0.4
	1 time a day	38	14.2
	2 times a day	217	81.0
	>2 times a day	12	4.5
How often do you floss?	Never	201	75.0
	Not every day	51	19.0
	1 time a day	14	5.2
	≥2 times a day	2	0.8
How often do you use interdental brushes?	Never	127	47.4
	Not every day	113	42.2
	1 time a day	26	9.7
	≥2 times a day	2	0.8
How often do you use woodsticks?	Never	249	92.9
	Not every day	17	6.3
	1 time a day	2	0.8
	≥2 times a day	0	0
What is your brushing duration?	<1 min	3	1.1
	1 min	39	14.6
	2 min	150	56.0
	3 min	52	19.4
	> 3 min	25	9.3
How often do you clean your tongue?	Never	135	50.4
	Sometimes	76	28.4
	Every day	57	21.3

Hybrid brushers using both the manual and power toothbrush (N = 15).

In order to assess the impact of tongue cleaning behaviour on tongue surface appearance, data are presented by those that never or sometimes (Figure 2a, 3a) clean their tongue and those that do so on a daily basis (Figure 2b, 3b). Results which were obtained by means of a Mann‐Whitney U test can be found in Table 4. The mean overall score for Tc and Td did not differ between those that cleaned or did not (regularly) do so (*P* = .17 and *P* = .10), respectively.

**Table 4:** Correlations and *P*‐values for the sumscores of tongue coating thickness (Tc) and discoloration (Td), for bleeding indices (BOMP and BOPP), plaque score index (PI) and age. Bonferroni correction: *α* < .0063 was accepted as being significant. Tongue cleaning behaviour and gender were assessed in relation to Tc, Td and bleeding indices (BOMP and BOPP) by Mann‐Whitney U test and corrected by Bonferroni correction: *α* < .007 considered as significant

Variables	Correlation coefficient	*P*‐value	95% CI
BOMP & Tc	.038	.540	−.082 to .166
BOMP & Td	.081	.186	−.039 to .213
BOPP & Tc	.032	.597	−.093 to .164
BOPP & Td	.122	.046	.021 to .271
PS & Tc	.13	.033	.016 to .254
PS & Td	.149	.015	.032 to .263
Age & Tc	.000	1.000	−.121 to .114
Age &Td	−.044	.475	−.157 to .068
Gender & Tc		.001	
Gender & Td		.001	
Tongue cleaning behaviour & Tc		.072	
Tongue cleaning behaviour & Td		.087	
Tongue cleaning behaviour & BOMP		.006	
Tongue cleaning behaviour & BOPP		.001	

Abbreviations: 95% CI = 95% Confidence Interval.

### 3.3 Gingival health

Table 4 shows all correlations between variables and Tc and Td, where also the *P*‐values and the 95% Confidence Intervals are given. Due to the multiple comparisons to explore correlation with the Spearman's test, a Bonferroni correction was applied. No correlation was found between BOPP or BOMP and Td or Tc.

Analyses of tongue cleaning behaviour showed that for BOMP as well as for BOPP a significant difference was found between those that cleaned on a daily basis or those that did not (regularly) do so. The mean BOMP scores were 0.41 and 0.49 respectively (*P* = .006) and the mean BOPP scores were 0.54 and 0.62 respectively (*P* = .001) (see also the boxplots in online  supplement Figure 2a,2b).

### 3.4 Dental plaque scores

Assessing the relationship between the dental plaque scores and tongue surface appearance showed no correlation between PI and Tc or Td. Analysis of tongue cleaning behaviour showed that for PI significant difference was found between those that cleaned or did not (regularly) do so.

### 3.5 Age

No correlation between Tc and Td with age was found.

### 3.6 Gender and menstrual cycle

When comparing female subjects with male subjects, a significant gender difference was found with respect to the Td and Tc (*P* = .001). Males tended to exhibit more discoloration and tongue coating thickness on the dorsum of the tongue. Considering that a difference exists between male and female participants when assessing Td, it was investigated whether hormonal changes are related to Tc or Td. Analysis was based on those participants who were aware of their menstrual cycle (N = 64). No significant differences were found between Td or Tc and the phases (3 different phases) of the menstrual cycle in the participant population existing of females (*P* = .55; *P* = .79 respectively).

## 4 DISCUSSION

The aim of this study was to evaluate various factors which may possibly be of influence on the surface appearance of the tongue regarding both coating (Tc) and discoloration (Td). Therefore, factors that purportedly are linked to tongue coating were investigated: gender, dental plaque,^7^ gingival inflammation,^13^, ^24^ age^3^ and tongue cleaning behaviour. When assessing the surface appearance of the tongue, most of the Tc and Td is found at the posterior 3 of the 9 sections of the tongue. This was also observed in other studies using the same tongue coating index.^3^, ^22^, ^25^ When comparing Tc and Td over the entire tongue surface, no significant correlation was found with gingivitis or dental plaque scores. Oral Hygiene Behavior, more in particular self‐reported tongue cleaning, as performed by the participants themselves did not appear to influence the observed Tc and Td. Male participants presented with a thicker tongue coating than female participants. However, those participants that reported daily tongue cleaning had on average a lower BOMP and BOPP score.

### 4.1 Tongue cleaning effect

No effect of tongue cleaning behaviour on Tc and Td was found (Table 4). One of the reasons for this may be that the highest level of Tc and Td was found at the posterior (middle) section of the tongue. Cleaning the posterior aspect of the tongue can be difficult due to a gagging reflex. Work from the past has shown that it is not possible to fully remove the micro‐organisms from the dorsum of the tongue after extensive use of a tongue scraper.^26^ More recently, it was confirmed that in patients with periodontitis, tongue cleaning does not influence the bacterial load on the tongue dorsum^27^ or in the saliva. Even though the same study showed that tongue coating was significantly less after 2 weeks of tongue cleaning.^27^

Tongue cleaning should therefore be performed on a daily basis due to the reformation process of the tongue coating.^28^ On the other hand, a controlled study has observed with professional instructions a significant reduction of tongue coating in healthy subjects before and after tongue cleaning.^29^ This suggests that an effect of Tc reduction can occur when clear instructions are given to subjects. The questionnaire, which was used for this study, only inquired about the frequency of tongue cleaning but did not particularly investigate which device was used for this. It can make a difference whether the tongue is cleaned with a brush of a scraper. For instance, a reduced gagging reflex has been observed with a tongue scraper compare to a brush.^30^ Therefore, a scraper could be helpful in more effectively cleaning the posterior aspect of the tongue. Although another study did not substantiate a difference in tongue surface appearance when comparing a scraper to a manual brush for tongue cleaning,^31^ both the scraper and brush reduced the amount of tongue coating (Tc) significantly. Tongue taste sensation ameliorated after 2 weeks of tongue cleaning, in particular for those using the scraper. Altogether it must be remembered that the assessment of oral hygiene habit was based on self‐report of the participants and may suffer from subjectivity in that desirable answers were provided.

Although no effect of tongue cleaning behaviour on Tc and Td was found, tongue cleaning on a daily basis was associated with slightly lower gingivitis (BOMP, BOPP) scores. This is in agreement with a recent study which concluded that tongue scraping can be taken into consideration in order to manage gingival inflammation.^32^ While subjects suffering from periodontal disease are more likely to have a thicker layer of coating compared to periodontally healthy controls.^3^ A possible biologic mechanism cannot be revealed by the data from the present study. Hypothetically, it could be that the visual aspects of the tongue surface do not change that much as a result of tongue cleaning, but it may result in a reduction of bacterial load which subsequently may have an impact on gingival health.

### 4.2 Age

The age of the individual influences Tc. In the age groups above 40 years, more thickness was observed than in the age groups younger than 40 years.^3^ This age‐related observation might be associated with changes in the nature of saliva of a decrease in salivary flow rate with increasing age.^33^ Also, a change of nutritional habits and the loss of dexterity to cope with oral hygiene could contribute to the tongue coating. Furthermore, there is an increase of filiform papillae with age.^5^ Considering this previous observation, in the current study a small age range was used with the intention to minimize this potential age effect. Indeed, this was not present for Tc and Td.

### 4.3 Hormonal changes

Hormonal fluctuations may influence the gingiva of pregnant women.^34^ To discover whether hormonal changes could affect the appearance of the tongue dorsum, the relation between the phase of the menstrual cycle and Tc and Td was examined. No association between hormonal changes and tongue coating could be confirmed, but only 64 women of the 118 women were aware of their menstrual phase which may have introduced an information bias.

Males tended to exhibit more discoloration and tongue coating thickness on the dorsum of the tongue. An explanation for this cannot be provided based on the current data. However, also in a recent publication, participants with a yellow tongue coating were more likely to be men.^35^ What may also play a role in our observation is that a fissured tongue was found to be more common in males.^36^ This aspect was not further assessed in this study but could be the subject of future observation.

### 4.4 Method of measurement

The procedure to assess tongue coating was a modification of the method as described by Miyazaki et al (1995).^3^, ^37^ In short, the tongue is visually assessed by one and the same trained examiner and divided in 9 more or less equal sections (3 anterior third, 3 middle third and 3 posterior third). Each section received a score for Tc (0‐2) and a score for Td (0‐4) as presented in as means in Table 1 and per Tc/Td score as percentage of subjects in Table 2. Over the years, a variety of different tongue coating indexes has been developed. The Winkel tongue coating index scores the tongue surface in 6 sections.^21^ Some studies have scored tongue coating as present or absent.^2^, ^38^ Whereas other studies ascribed the coatings as none, light, medium or heavy.^39^, ^40^, ^41^ Furthermore, several studies described an index with scores 0‐4, making a distinction between the size of the covered area and the thickness of the coating.^42^, ^43^, ^44^, ^45^ Other methods of assessing the tongue coating are more complex such as digital imaging analysis^46^ or wet weight analysis,^1^, ^6^, ^47^ and these tend to be more precise.^48^ Moreover, the assessment of coating can also be performed using autofluorescence^49^ or utilize bacterial counts (expressed as cfu cm^2^).^50^ However, complex methods like these are not easily applied in routine clinical practice. The method used in the present study is a simple method for clinical use, where no complex instruments are required and which can be completed in less than one minute.^3^, ^37^ It is therefore also convenient in clinical practice.^51^ Reproducibility of a clinical scoring method was found to be moderate to strong and varies between 0.48‐0.84.^49^ To establish higher reliability, the limitations of the subjectivity of the assessor can be overcome through calibration training.^51^ Also, reliability increases when criteria are simplified.^52^ With respect to analysis of tongue coating scores, various ways have been published. Winkel et al^21^ and van der Sluijs et al^22^ used the sum of all scores, Mantilla Gómez et al^3^ used the highest score and Kim et al^53^ used the mean score while Kobayashi et al^54^and Shimizu et al^48^ calculated an index. For the present study, we adhered to the most common method as proposed by Winkel et al^21^ and van der Sluijs et al^22^

### 4.5 Limitations of the study

The specific population examined in this study, which consisted of healthy young adults, introduces a limitation in generalizability. Furthermore, research on the topic of tongue cleaning and the effect it may have on the tongue surface appearance should not only consist of a cross‐sectional investigation but could also be explored in a controlled study design.

Tongue coating correlates with soft food intake.^31^ Also, coffee increases the amount of coating^11^ and discoloration.^3^ This was however not assessed in the current evaluation.

## 5 CONCLUSION

BOMP, BOPP or PI score did not appear to be linked to Tc and Td. A significant gender difference was found for Tc and Td. Self‐reported tongue cleaning behaviour was associated with slightly lower BOMP and BOPP scores.

## 6 CLINICAL RELEVANCE

### 6.1 Scientific rationale for the study

Tongue coating may affect the oral microflora and in effect plaque accumulation and gingival inflammation. Tongue cleaning may affect tongue surface appearance.

### 6.2 Principal findings

There was no correlation between gingivitis or plaque scores and tongue coating thickness or discoloration. Self‐reported tongue cleaning did not show to have an effect on both coating thickness and discoloration; however, it was associated with lower gingivitis scores.

### 6.3 Practical implication

The observed relationship between BOMP or BOPP and tongue cleaning indicates that this may be an oral hygiene aspect that deserves further investigation.

## CONFLICT OF INTEREST

The authors declare no conflict of interest to this study.

## AUTHOR CONTRIBUTIONS

LM van Gils: contributed to the analysis and interpretation of data and drafted the manuscript. DE Slot: contributed to conception and design, search and selection, analysis and interpretation and critically revised the manuscript. E. Van der Sluijs: contributed to the design and conduct of this study, and critically revised the manuscript. NL Hennequin‐Hoenderdos: contributed to the design and conduct of this study, and critically revised the manuscript. Fridus (GA) Van der Weijden: contributed to the conception and design, analysis and interpretation, and drafted the manuscript. All authors gave final approval and agreed to be accountable for all aspects of work ensuring integrity and accuracy.
