# Supplementary material for: Tongue coating in relationship to gender, plaque, gingivitis and tongue cleaning behaviour in systemically healthy young adults
Source: Int J Dent Hyg. 2019 Oct 10;18(1):62–72. doi: 10.1111/idh.12416 (PMC7004167; doi:10.1111/idh.12416)

**Online appendices**

**Anterior**

**Right**

**Left**

**Posterior**

LP

MP

RP

LM

MM

RM

RA

MA

LA

Figure S1. *Dorsum of the tongue divided in 9 sections.* The tongue division as described by Gomez et al. (2001). From tip to back as anterior (A), middle (M) and posterior (R).

| Table S1a *Description of clinical criteria per score of the Dutch periodontal screening index (DPSI), to apply per sextant based on the site with the highest score by Mantilla Gomez^5^.* | |
| --- | --- |
| Scores | Characteristics |
| 0 | no pockets deeper than 3mm  no bleeding on probing  no calculus and/or overhanging restorations |
| 1 | the same criteria as for score 0, but bleeding on probing |
| 2 | the same criteria as for score 1, but with calculus and/or overhanging restorations |
| 3- | pockets from 4-5 mm  bleeding on probing  supra and subgingival calculus and/or overhanging restorations  no recession |
| 3+ | the same criteria as for score 3-, but with recession |
| 4 | pockets deeper than 6 mmd |
| There must be at least two teeth in each sextant, if there is only one tooth, it will be counted in the adjacent sextant. | |

| Table S2b *Gingival inflammation assessed by scoring BOMP* ^15 ~~21~~^ *and BOPP^15^* ^~~45~~^*.* | |
| --- | --- |
| 0 | No bleeding |
| 1 | Bleeding |
| 2 | Excessive bleeding |
| BOMP = Bleeding On Marginal Probing  BOPP = Bleeding On Pocket Probing | |

| Table S3 *Values of correlation coefficients for interpretation^~~23~~^* ^20^*.* | |
| --- | --- |
| .00 ─ .19 | Very weak |
| .20 ─ .39 | Weak |
| .40 ─ .59 | Moderate |
| .60 ─ .79 | Strong |
| .80 ─ 1.0 | Very strong |

Figure S2a. *Distribution in percentages of thickness of tongue surface coating scores according to Mantilla Gómez separated for each of the 9 sections of the tongue^3^* (see online figure 1 and table 3) *for only those participants who mentioned being categorized as “never or sometimes tongue cleaners”* (N = 211).

^3^See footnote figure 1a.

Figure S2b. *Distribution in percentages of thickness of tongue coating scores according to Mantilla Gómez separated for each of the 9 sections of the tongue^4^* (see online figure 1and table 3.) *for “daily tongue cleaners”* (N = 57).

^4^See footnote figure 1a.

Figure S3a. *Distribution in percentages of tongue surface discoloration scores according to Mantilla Gómez separated for each of the 9 sections of the tongue^5^* (see online figure 1. and table 3.) *for only those participants who mentioned being categorized as “never or sometimes tongue cleaners”* (N = 211).

^5^See footnote figure 1a.

Figure S3b*.* *Distribution in percentages of tongue surface discoloration scores according to Mantilla Gómez separated for each of the 9 sections of the tongue^6^* (see online figure 1. and table 3.) *for “daily tongue cleaners”* (N = 57).

^6^See also footnote figure 1a.

~~Figure S4a.~~ *~~Scatterplot of age and tongue coating thickness scores.~~*


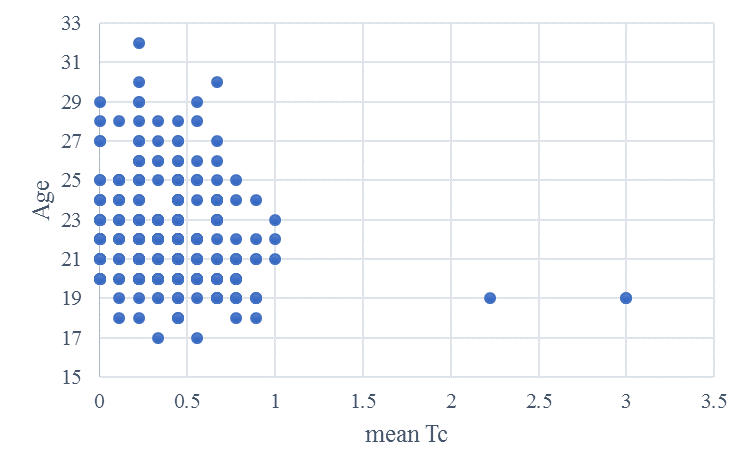


~~Figure S4b.~~  *~~Scatterplot of age and tongue surface discoloration scores.~~*


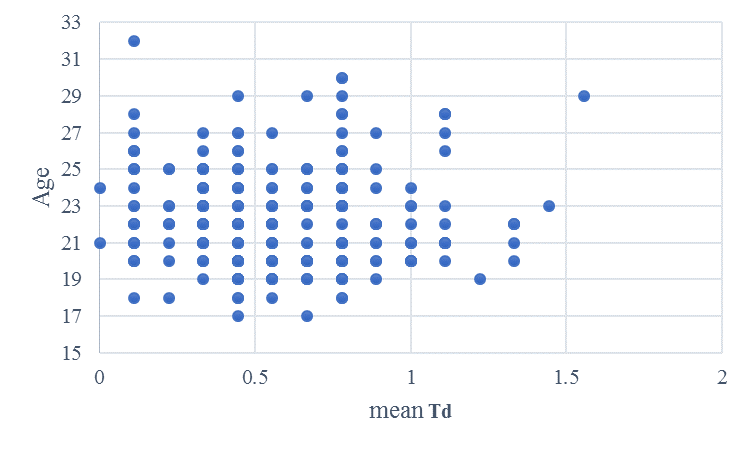


~~Figure S5a.~~ *~~Scatterplot of mean BOMP scores and mean tongue coating thickness scores.~~*

~~Figure S5b.~~ *~~Scatterplot of mean BOPP scores and mean tongue coating thickness scores.~~*

Figure S4a. Boxplot of tongue cleaning behavior, daily or sometimes and never, and the mean scores of BOMP.


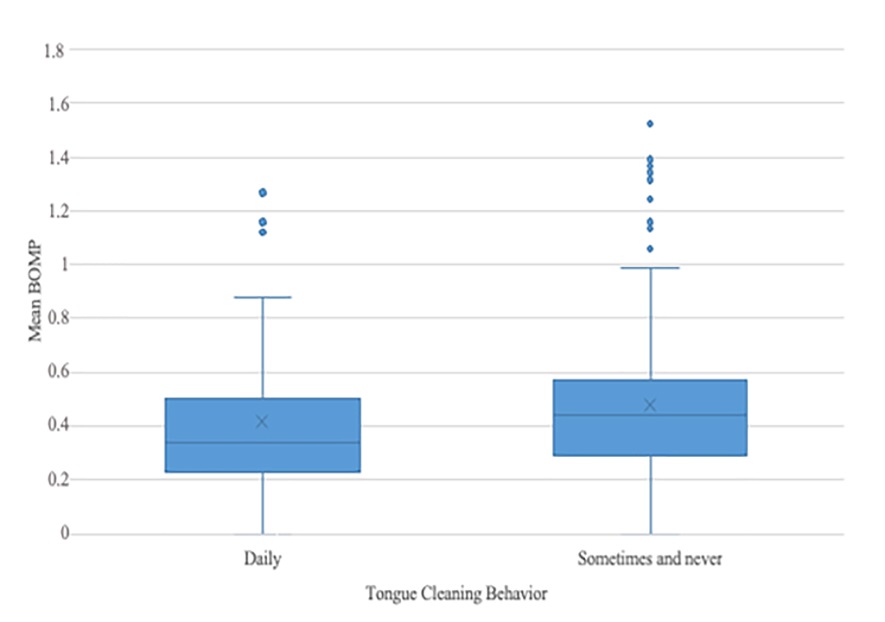


Figure S4b. Boxplot of tongue cleaning behavior, daily or sometimes and never, and the mean scores of BOPP.


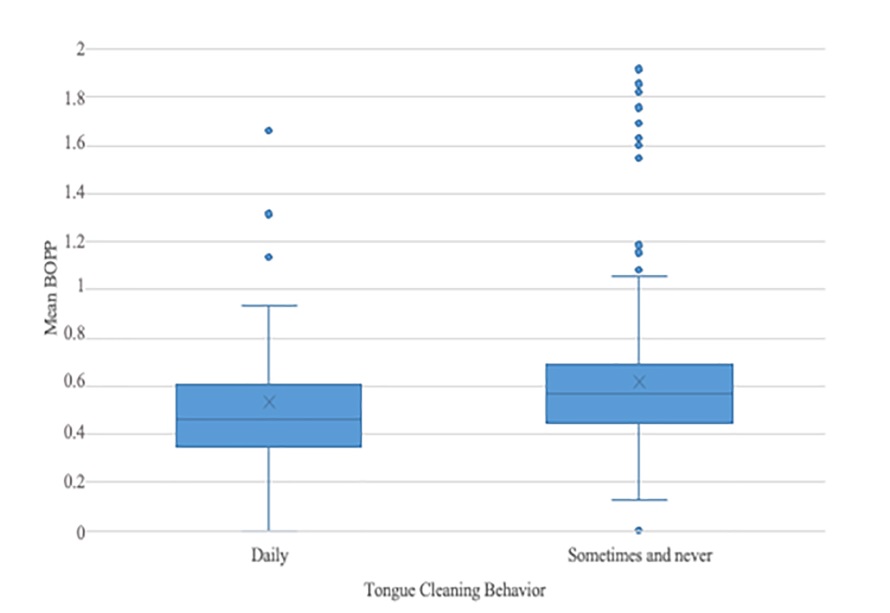

Supplement: Supplementary file 1 [file IDH-18-62-s001.docx]
